# Bullet embolization from the right subclavian artery to the right common femoral artery

**DOI:** 10.1093/jscr/rjad606

**Published:** 2023-10-31

**Authors:** Nahar Alselaim, Sultanah Bin Gheshayan, Moneera Bin Saleem, Abdullah Alwahbi

**Affiliations:** Department of General Surgery, King Abdulaziz Medical City, Ministry of National Guard Health Affairs, Khashem Al Aan, Prince Jaber Al Sabah Street, Riyadh 11426, Saudi Arabia; College of Medicine, King Saud bin Abdulaziz University for Health Sciences, Khashem Al Aan, Prince Jaber Al Sabah Street, PO Box 22490, Riyadh,11426, Saudi Arabia; King Abdullah International Medical Research Center, Khashem Al Aan, Prince Jaber Al Sabah Street, Riyadh 11426, Saudi Arabia; Department of General Surgery, King Abdulaziz Medical City, Ministry of National Guard Health Affairs, Khashem Al Aan, Prince Jaber Al Sabah Street, Riyadh 11426, Saudi Arabia; College of Medicine, King Saud bin Abdulaziz University for Health Sciences, Khashem Al Aan, Prince Jaber Al Sabah Street, PO Box 22490, Riyadh,11426, Saudi Arabia; King Abdullah International Medical Research Center, Khashem Al Aan, Prince Jaber Al Sabah Street, Riyadh 11426, Saudi Arabia; Department of Surgery, King Abdullah bin Abdulaziz University Hospital, Princess Nourah Bint Abdulrahman University, Airport Road, King Khalid International Airport, Riyadh 11564, Saudi Arabia; Department of Vascular Surgery, King Abdulaziz Medical City, Ministry of National Guard Health Affairs, Khashem Al Aan, Prince Jaber Al Sabah Street, Riyadh 11426, Saudi Arabia; College of Medicine, King Saud bin Abdulaziz University for Health Sciences, Khashem Al Aan, Prince Jaber Al Sabah Street, PO Box 22490, Riyadh,11426, Saudi Arabia; King Abdullah International Medical Research Center, Khashem Al Aan, Prince Jaber Al Sabah Street, Riyadh 11426, Saudi Arabia

**Keywords:** gunshot, penetrating trauma, bullet embolism, subclavian artery, femoral artery, arteriotomy

## Abstract

Bullet embolization from a gunshot wound is a rare entity in trauma patients. We report a case of a 37-year-old female patient who was brought to the trauma unit after sustaining multiple gunshots to the chest and abdomen. Followed by embolization of the bullet from the right subclavian artery to the right common femoral artery. Had successful retrieval of the bullet via a transverse arteriotomy.

## Introduction

A rare complication of gunshot wound injuries is bullet embolization [1]. Once the bullet gains access to the intravascular system, many factors other than the blood flow can influence its migration, and it can result in serious complications, from limb ischemia to life-threatening conditions. More commonly, the bullet is found post-mortem. Those cases often pose a significant diagnostic and therapeutic challenge. Here, we describe a puzzling case where a wandering bullet made its path.

## Case report

**Figure 1:**
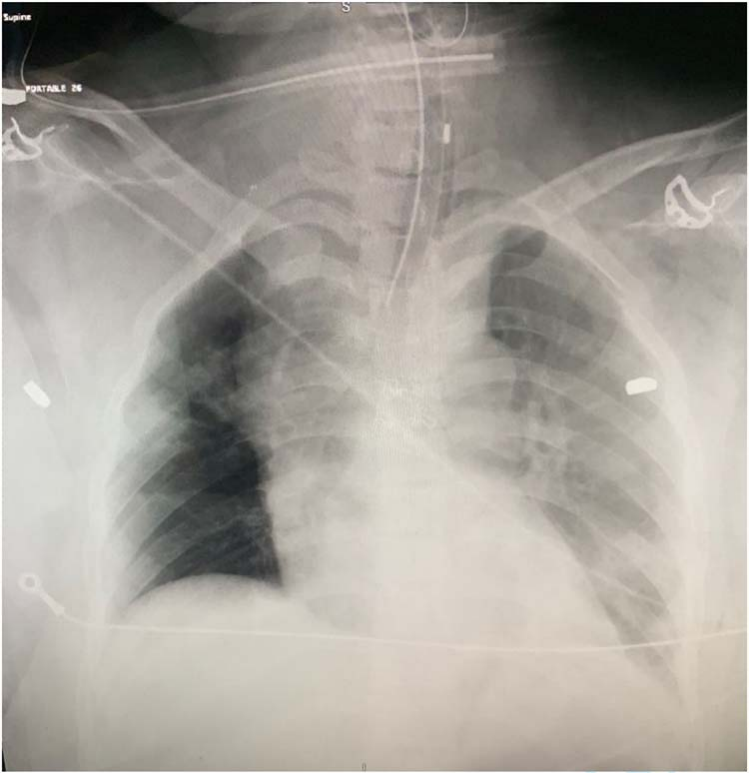
Chest radiograph showing bullet fragments in the chest cavity.

A 37-year-old female was brought to the trauma unit after sustaining multiple gunshots to the chest and abdomen. She was alert, conscious, and hemodynamically stable. The first responders mentioned that the patient was complaining of severe right lower limb pain at the scene. Physical examination revealed a gunshot wound in the suprasternal area with hematoma. Therefore, the patient was intubated to protect her airway. Several gunshot wounds were identified over the anterior aspect of her body, including bilateral sides of her head, central neck zone 1, right second intercostal space at mid clavicular line, and right upper quadrant of her abdomen. On the posterior aspect, two wounds over the right paraspinal area and another over the subcapsular area. She had diminished distal pulses in the right lower limb. Her chest x-ray revealed three bullet fragments: one adjacent to the right acromioclavicular junction in the right scapula and another projecting over the left scapula. Bilateral chest tubes were inserted. Extended focused assessment sonography for trauma was negative (Fig. 1).

**Figure 2:**
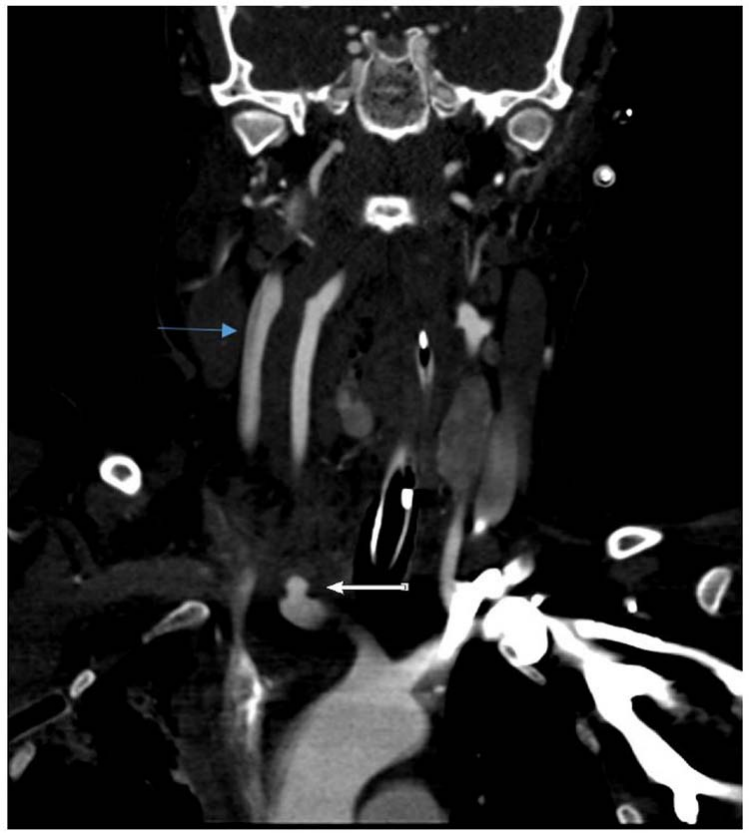
Right common carotid intimal flap (upper arrow) and pseudoaneurysm at origin (lower arrow).

**Figure 3:**
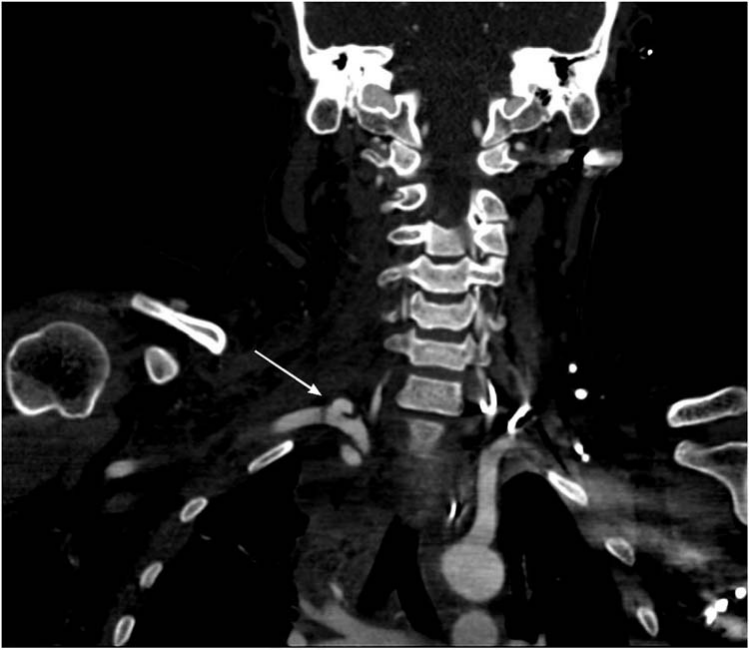
Right subclavian pseudoaneurysm.

She remained hemodynamically stable; therefore, the patient was transferred to Computed tomography (CT) scan for optimal preoperative planning. Head and neck CT showed right common carotid dissection with a small pseudoaneurysm (Fig. 2). And bullet fragment causing proximal filling defect measuring 1.4 cm long without active extravasation. Surrounding the right common carotid was a hematoma extending to the anterior neck and superior mediastinum. Also, Right subclavian dissection with three pseudoaneurysms (Fig. 3) proximal and distal to the right vertebral artery takeoff, the largest measures 1.1 × 0.8 cm. Multiple scattered metallic fragments were also noted at the subcutaneous tissue of bilateral temporal bones and the occipital area.

**Figure 4:**
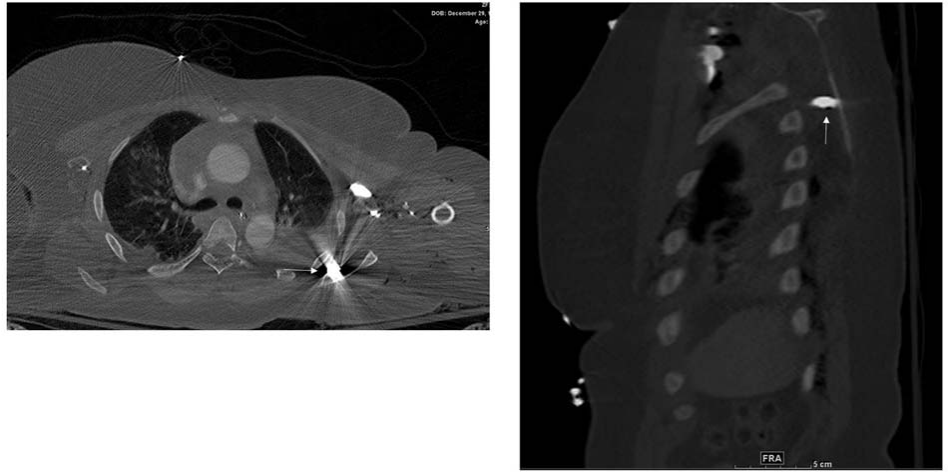
Bullet fragments adjacent to the right acromioclavicular junction and posterolateral.

Chest CT showed three bullet fragments. One was adjacent to the right acromioclavicular junction, another was posterolateral to the right periscapular muscles, and the third was anterior to the left scapula (Fig. 4). In addition to right lung contusions, left fifth rib fracture with pneumothorax, and bilateral pleural hemothorax more at the left side.

**Figure 5:**
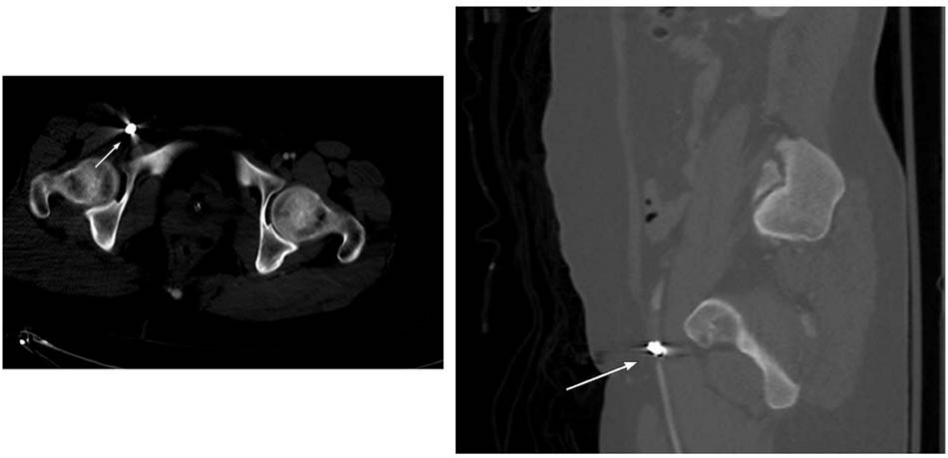
Right distal external iliac artery bullet embolus.

**Figure 6:**
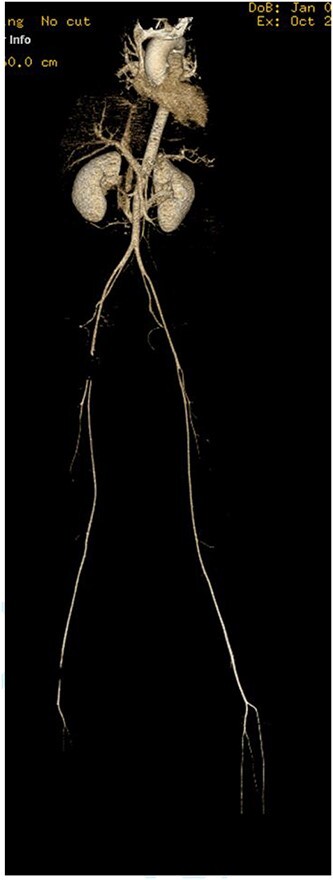
Filling defect in the external iliac artery with no extravasation and the contrast passing to the superficial femoral arteries.

Abdomen and pelvis CT showed an intra-abdominal bullet fragment abutting the anterior gastric wall without gastric injury and a minor splenic laceration. Also, a bullet fragment in the right distal external iliac artery at the level of bifurcation (Fig. 5) causing a filling defect, however no extravasation, and the contrast passed to the superficial femoral arteries (Fig. 6).

**Figure 7:**
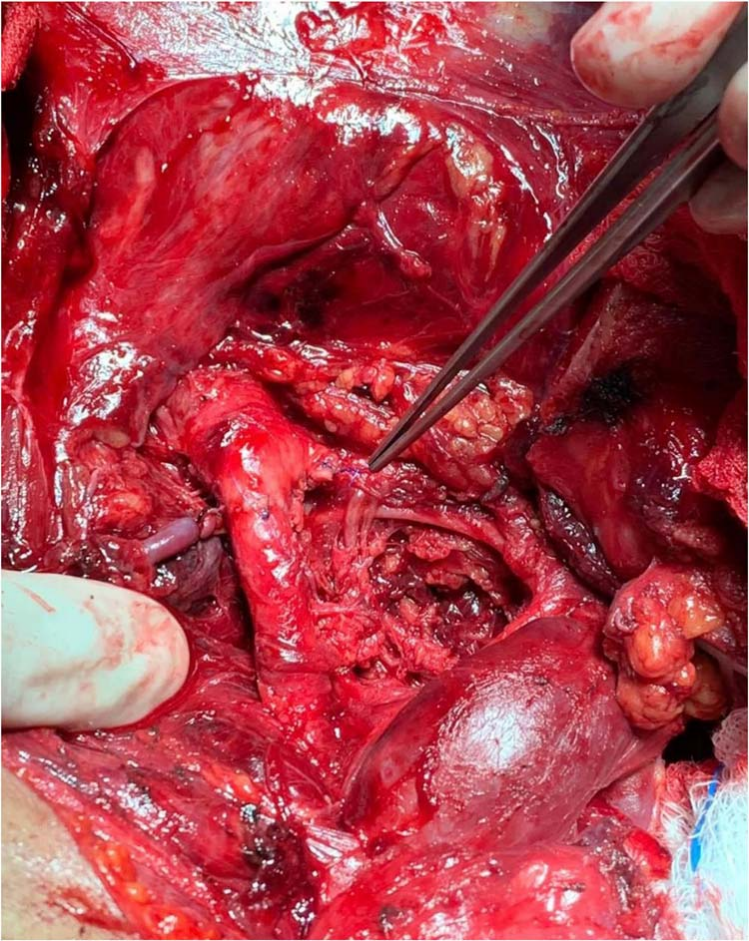
The right common carotid artery end to end anastomosis.

**Figure 8:**
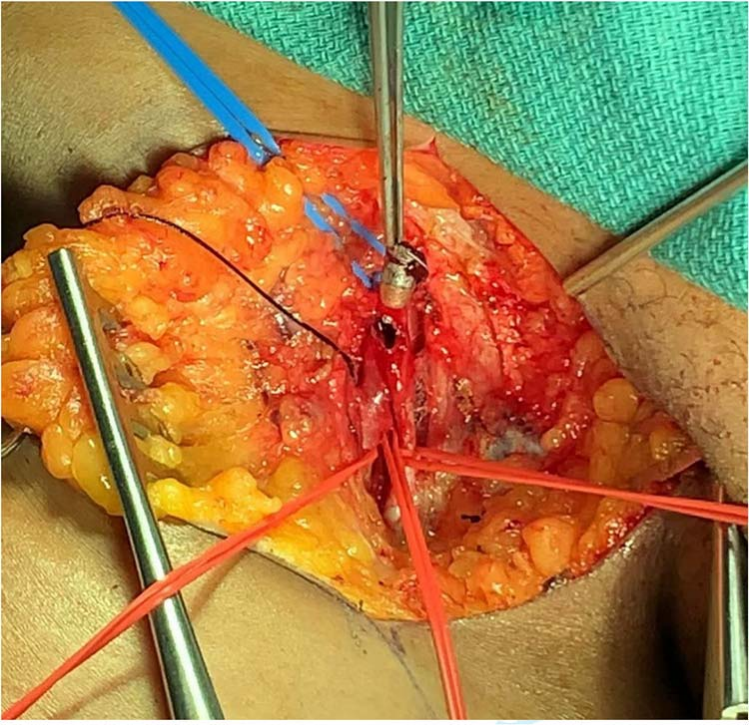
Bullet extraction from right common femoral artery.

The patient was taken to the operating room. A median sternotomy was performed, which revealed extensive injuries to the right carotid and subclavian arteries with active bleeding. The innominate artery was controlled, followed by the distal ends of the injured right common and subclavian arteries. The arteries were severely damaged by bullets. The right common carotid artery was repaired with end to end anastomosis, while the subclavian artery was ligated due to the extensive tissue loss and as a damage control maneuver given the instability of the patient (Fig. 7). Then the right groin was explored, and the common femoral, deep, and superficial femoral arteries were controlled. A transverse arteriotomy was performed, which showed the bullet embolized at the common femoral bifurcation (Fig. 8). The bullet was extracted, and the arteriotomy was closed. Exploratory laparotomy revealed no intra-abdominal injuries, and a bullet was found anterior to the peritoneum, which was extracted. The patient had multiple shrapnel in the occipital area, which were removed surgically, and the wounds were debrided. Afterward, she was transferred to the intensive care unit for postoperative care. Six weeks after the injury, the patient was discharged home.

## Discussion

Bullet embolus is a rare but well described occurrence that is encountered among some of the gunshot victims. It was first described in 1834 by Thomas Davis, and since then, several reports have been published describing bullet embolus [2]. The diagnosis, in many instances, is not straight-forward and requires a high index of suspension, given that 20% of patients with bullet embolus are asymptomatic. Many emboli could be easily missed since a large percentage of these patients will have other distracting injuries that require immediate attention, which could delay the recognition of such injuries. Signs and symptoms of limb ischemia, unequal number of exit and entrance wounds, and vascular injuries could indicate possible bullet embolus. Bullet emboli are more common in the arterial than the venous system. Bullet migration is usually immediate. However, in one of the reported cases in the literature, it can take up to 14 years [1].

Penetrating injury to major vessels could be fatal; however, in some cases, a bullet may lose its kinetic energy at the entrance, allowing it to penetrate the vessel but not transfix it. Thereby entering the vascular system and as a result, it travels through the bloodstream until it occlude a peripheral artery in a distant location from where the injury occurred [3]. This explains the scenario in our case where the bullet’s kinetic energy was sufficient to penetrate the subclavian artery but not enough to exit it.

The embolization of bullets and other projectiles can occur due to various factors, such as the size and shape of the fragment. It occurs more frequently with a small caliber, usually a low-velocity bullet [4]. Other contributing factors include the size and angle of the vessel, the body’s position, gravity’s effect, muscular and respiratory movements [5–7].

There are multiple pathways through which a bullet might migrate. Arterial and venous bullet emboli generally follow the direction of blood flow. However, in some cases, bullet emboli can travel in a retrograde fashion, where the bullet moves against the direction of blood flow by gravity [8]. Rare cases of paradoxical embolization can occur, where the object moves from the venous circulation and subsequently gains access to the arterial circulation through a congenital or traumatic heart defect [9]. In our case, an echocardiogram was done for this patient and came back negative for any traumatic or congenital anatomical anomalies, which exclude paradoxical embolization. And the fact that the patient did not develop a pulmonary embolism makes it highly unlikely that the bullet made its way back into the heart. In this case, the bullet probably traveled retrograde back into the aorta, only reaching the right common femoral bifurcation.

The Vietnam Vascular Registry analyzed ~7500 cases and reported that arterial missile emboli occurred more frequently than venous emboli (82 vs 18%) [10]. The site of impaction usually occurs at vascular bifurcations. The incidence of embolism to the left lower extremity was higher than the right due to a more acute angle between the left common iliac artery and the aorta [11].

Management options mainly depend on the symptoms and location of the bullet. Endovascular extraction may be an option in symptomatic cases if the patient’s clinical condition permits. However, emergency surgery is often required, particularly if there is arterial embolization [12].

In asymptomatic patients, the management plan is not clearly defined. In selected cases, asymptomatic patients can be managed conservatively if the risk of the procedure outweighs the potential benefits of bullet extraction [13].

In conclusion, bullet embolization to the lower extremities is a rare entity.

Arterial embolism should be taken into consideration when there is no evidence of an exit wound. Therefore, careful and thorough physical and radiological exams are essential. A management plan for such cases should be individualized for each patient.

## Data Availability

The authors have verified that the data supporting the study can be found in the article and its supplementary materials.
